# Role of the Retromer Complex in Neurodegenerative Diseases

**DOI:** 10.3389/fnagi.2016.00042

**Published:** 2016-03-01

**Authors:** Chaosi Li, Syed Zahid Ali Shah, Deming Zhao, Lifeng Yang

**Affiliations:** National Animal Transmissible Spongiform Encephalopathy Laboratory, Key Laboratory of Animal Epidemiology and Zoonosis of Ministry of Agriculture, College of Veterinary Medicine and State Key Laboratory of Agrobiotechnology, China Agricultural UniversityBeijing, China

**Keywords:** retromer complex, sorting nexin family member 27, Wiskott-Aldrich syndrome protein and scar homolog, Alzheimer’s disease, Parkinson’s disease

## Abstract

The retromer complex is a protein complex that plays a central role in endosomal trafficking. Retromer dysfunction has been linked to a growing number of neurological disorders. The process of intracellular trafficking and recycling is crucial for maintaining normal intracellular homeostasis, which is partly achieved through the activity of the retromer complex. The retromer complex plays a primary role in sorting endosomal cargo back to the cell surface for reuse, to the trans-Golgi network (TGN), or alternatively to specialized endomembrane compartments, in which the cargo is not subjected to lysosomal-mediated degradation. In most cases, the retromer acts as a core that interacts with associated proteins, including sorting nexin family member 27 (SNX27), members of the vacuolar protein sorting 10 (VPS10) receptor family, the major endosomal actin polymerization-promoting complex known as Wiskott-Aldrich syndrome protein and scar homolog (WASH), and other proteins. Some of the molecules carried by the retromer complex are risk factors for neurodegenerative diseases. Defects such as haplo-insufficiency or mutations in one or several units of the retromer complex lead to various pathologies. Here, we summarize the molecular architecture of the retromer complex and the roles of this system in intracellular trafficking related the pathogenesis of neurodegenerative diseases.

## Introduction

**Figure 1:**
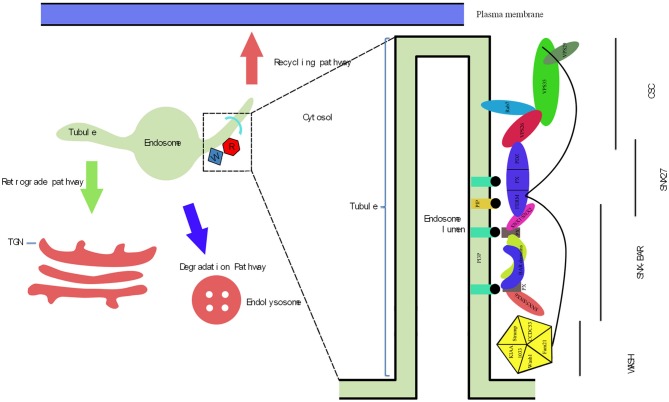
**The endosomal trafficking managed by retromer and the molecular architecture of inter-relationship between the retromer complex, sorting nexin family member 27 (SNX27) and Wiskott-Aldrich syndrome protein and scar homolog (WASH) complex.** (1) There are three trafficking pathways regulated by retromer. Initially, the retrograde pathway, cargo proteins are retrieved from the endosomes and trafficked to the trans-Golgi network (TGN). The second is the recycling pathway in which cargo proteins are trafficked to the plasma membrane (PM) for reuse. The first two transport routes above both via tubules depended that extend out of edosomal membranes. The last pathway is degradative pathway, which helps in internalizing cargoes to the endosome then gradually matures to become the multi-vesicular late endosome (LE) before fusing to existing lysosomes resulting in the degradation of cargoes in the endolysosome. (2) The endosomal-sorting complex is composed of retromer complex, SNX27 and WASH complex. The WASH complex is comprised of five proteins: KIAA1033, Strumpellin, FAM21, WASH1, and Coiled-Coil Domain Containing 53 (CCDC53). SNX27 is composed of protein-interaction domains often found in multi-domain scaffolding proteins known as PDZ domain, the phoxhomology (PX) domain and the FERM domain. Retromer complex consist of cargo-selective complex (CSC; VPS35, VPS26 and VPS29) and SNX-BAR (SNX1 or SNX2 and SNX5 or SNX6). VPS35 supports a platform that VPS26 and VPS29 bind to it. Rab7 interacts with VPS26 and VPS35 as well as recruits the CSC to the endosome membrane. VPS26 binds to the PDZ domain of SNX27. The FERM-like domain of the SNX27 directly interacts with SNX1 or SNX2 of SNX-BAR. The recruitment of SNX27 and SNX-BAR to endosomes is both through the binding of phosphatidylinositol-3-phosphate (PI3P) by their PX domain. In addition, within the FERM domain of SNX27, is a second site with high affinity for PIP. As for the WASH complex, which is responsible to link itself to the VPS35, VPS29 and SNX27 FERM domain through its unstructured C-terminal tail in FAM21 and is recruited by CSC.

Cargo originating from the plasma membrane (PM) and biosynthetic pathways is internalized to endosomes through endocytosis, after which cargo proteins are recruited to different destinations for many cellular activities. Internalized nutrients are targeted to different organelles for processing, pathogens are transported into lysosomes for degradation, and newly synthesized proteins are transported to specific cellular locations for specific functions (Lakadamyali et al., 2006). The cargo sorting retromer complex, a membrane coat complex that is a crucial mediator of endosomal protein sorting, was first identified in yeast as a peripheral membrane protein complex (Seaman, 2004). Internalized cargoes are sorted by the retromer complex in three different ways (Figure 1). First, in the recycling pathway, proteins such as nutrient transporters, mitogenic signaling receptors, and cell adhesion receptors are recycled from endosomes to the PM for reuse (Maxfield and McGraw, 2004; Hsu et al., 2012). The second pathway is a retrograde pathway in which cargo is trafficked from the endosomes to the trans-Golgi network (TGN). The principal cargoes of the retrograde pathways are sorting receptors, soluble N-ethylmaleimide-sensitive factor attachment protein receptors (SNAREs), and other molecules with functions dependent on continual retrieval from the endosomal system and return to biosynthetic pathways (Bonifacino and Rojas, 2006; Johannes and Popoff, 2008). The third pathway is a degradative pathway that involves internalization of cargoes on the PM into early endosomes (EEs), which gradually mature into multi-vesicular late endosomes (LEs) and fuse to existing lysosomes, resulting in degradation of endolysosomal cargo (Cullen and Korswagen, 2012). The retromer complex recruits membrane cargoes from the vacuolar domain to retromer-decorated tubular domains for retrieval and recycling; such cargoes can be selectively trafficked to avoid transportation to LEs and subsequent degradation (Lu and Hong, 2014). Therefore, the retromer complex plays a role in limiting lysosome-mediated turnover. During this process, the retromer complex recruits additional proteins that capture and package cargoes and direct them to alternate sorting fates. Normally, there is a coordinated relationship between the recycling, retrograde, and degradative pathways, which maintains cellular metabolic balance and homeostasis. However, if any of the above-mentioned pathways are hampered by events such as deregulated protein processing or missorting of intracellular proteins within endosomal–lysosomal pathways, deficiencies in cargo sorting, organelle development, and signal transduction can occur, sometimes leading to protein misfolding disorders (PMDs), including Alzheimer’s disease (AD), Parkinson’s disease (PD), frontotemporal lobar degeneration (FTLD), and Down’s syndrome (Reitz, 2015).

## Retromer and Its Partner Proteins

### Sorting Nexin Bar (SNX-BAR)

Retromer is composed of two sub-protein complexes. In addition to the cargo-selective complex (CSC), sorting nexins (SNX) form a hetero-dimer complex composed of vacuolar protein sorting 5 (VPS5) and VPS17 in yeast (Seaman et al., 1998). SNX proteins differ significantly between species. In metazoans, SNX-BAR family members include SNX1, SNX2, SNX5, and SNX6. SNX1 and SNX2 are VPS5 orthologs that originated from gene duplication in a vertebrate ancestor; however, SNX5 and SNX6 resulted from duplication of VPS17 in a metazoan ancestor (Harterink et al., 2011). All SNXs include two membrane-binding domains, a phox homology (PX) domain and Bin/Amphiphysin/Rvs domain (BAR), and are thus termed SXN-BAR proteins. The BAR domain resides at the C-terminal of SNX proteins. The PX domain is capable of sensing, formatting, and stabilizing membrane curvature by binding to membrane surfaces, leading to endosomal budding in yeast and mammalian cells (Carlton et al., 2004; Frost et al., 2009; van Weering et al., 2012). SNX-BAR dimers associate with the membrane independently of the CSC in mammalian cells (Seaman, 2004). SNX-BAR recruitment is mediated through binding to phosphatidylinositol 3-phosphate (PtdIns(3)P) in EEs in yeast and mammalian cells (Figure 1; Gillooly et al., 2000; Cozier et al., 2002; Harterink et al., 2011; Vardarajan et al., 2012). In LEs, phosphatidylinositol 3,5-bisphosphate (PtdIns(3,5)P2) is bound by the PX domain of SNX-BAR dimers in yeast and mammalian cells (Gillooly et al., 2000; Cozier et al., 2002; Huotari and Helenius, 2011; Jean and Kiger, 2012).

In addition to mediating retrograde transport between endosomes and the TGN, the SNX-BAR coat complex transports other molecules through tubular-based endosomal trafficking (Cullen, 2008). For example, SNX1 interacts with the tails of G-protein-coupled receptors (GPCRs) *in vitro* and traffics the GPCR protease-activated receptor-1 (PAR1) to the lysosome for degradation. In addition, SNX1 regulates recycling of the P2Y1 receptor, a GPCR, via a slow recycling pathway in mammalian cells (Wang et al., 2002; Heydorn et al., 2004; Nisar et al., 2010).

### Cargo Selective Complex

In mammalian cells, the CSC is a highly conserved hetero-trimer subcomplex consisting of VPS35, VPS26, and VPS29. The CSC evolved in a common eukaryotic ancestor. Among the three components of the CSC, VPS35 acts as the key component, providing linkage between VPS26 and VPS29 (Figure 1). Mammals express two VPS26 orthologs, VPS26A and VPS26B (Burd and Cullen, 2014), which possess an arresting fold and bind to the highly conserved amino-terminal region of VPS35 (Collins et al., 2005; Shi et al., 2006). However, an L108P mutation within the PRLYL conserved assembly motif of VPS35 blocks binding of VPS35 to VPS26 in yeast and mammalian cells (Gokool et al., 2007). VPS29 possesses a metallophosphoesterase fold and binds to the carboxy-terminal of VPS35, where it is proposed to bind with the helical solenoid of VPS35 in mammalian cells (Wang et al., 2005; Rojas et al., 2008; Norwood et al., 2011). The loss of function of the D620N VPS35 mutant was not as pronounced as that of the H675R mutant, which cannot bind VPS29 in yeast or mammalian cells (Zhao et al., 2007; Zavodszky et al., 2014). VPS35, VPS26, and VPS29 form a trimeric complex that does not have intrinsic membrane-binding activity. Retromer recruitment is mediated by RAS-related GTP-binding proteins (RABs). In humans, Rab7 (Ypt7 in yeast) binds to LEs via direct interaction with N-terminal conserved regions in VPS35 and VPS26. In addition, amino-terminal residues (189–252) of VPS35 are essential for Rab7-mediated retromer recruitment in mammalian cells (Priya et al., 2015). In mammals, the binding sites of VPS35 and VPS26 on Rab7 are near each other (Priya et al., 2015). Importantly, association of VPS26 with VPS35 results in high affinity binding between the CSC and activated Rab7 (Priya et al., 2015). TBC1D5, a putative Rab GTPase-activating protein for Rab7, inhibits recruitment of the retromer CSC in mammalian cells (Rojas et al., 2008; Seaman et al., 2009; Vardarajan et al., 2012).

However, in EEs, the CSC is recruited through SNX3 bound to the membrane, where the retromer complex subsequently transports integral membrane retrograde cargo (Harrison et al., 2014). It would naturally follow that many membrane proteins require retromer for their localization; indeed, a recent proteomic study identified more than 100 proteins that were depleted from the cell surface following loss of retromer function in mammalian cells (Steinberg et al., 2013). In addition to sorting cargoes through direct binding, CSC also sorts cargoes by recruitment of accessory proteins, such as the Wiskott-Aldrich syndrome protein and scar homolog (WASH) complex, as well as binding of SNX27 with cargoes (Gomez and Billadeau, 2009; Harbour et al., 2010).

### Sorting Nexin 27 (SNX27)

SNX27 is composed protein-interaction domains often found in multi-domain scaffolding proteins, including a PDZ domain, PX domain, and FERM domain (Figure 1). The PX domain is a phosphoinositide-binding domain that is conserved in yeast and humans. The FERM domain, originally identified in rats as an alternative-splicing product of the methamphetamine responsive transcript 1 (Mrt1) gene, is a widespread cytoskeleton protein module involved in localizing proteins to the PM in mammalian cells (Kajii et al., 2003). In an arrangement unique among PX domain proteins, the N-terminal PDZ domain of SNX27 is upstream of the PX domain; three sub domains, F1, F2, and F3, constitute the FERM-like domain at the C-terminal region of SNX27 in mammals (Ghai et al., 2011). Two associations with the SNX-BAR-retromer complex are displayed independently by SNX27. In addition to direct interaction of the FERM-like domain with SNX1 (and likely SNX2), the PDZ domain directly binds to VPS26, whereas the FERM-like domain combines with the unstructured C-terminal tail of FAM21, an unit of the WASH retromer accessory protein complex, in mammalian cells (Figure 1; Ghai et al., 2011).

In the pathway from endosomes to the PM, SNX27 acts as a retromer adapter and a major regulator of transport via binding with PDZ-binding motifs in mammalian cells (Cao et al., 1999; Temkin et al., 2011; Steinberg et al., 2013). In this pathway, the critical step is the recruitment of SNX27 to endosomes through binding of phosphatidylinositol-3-phosphate (PI3P) by the PX domain in T-cells (Ghai et al., 2015). In addition, the FERM domain contains a second site with high affinity for PtdInsP species, which are enriched at the PM and within LE compartments in T-cells. In other words, perturbing the interaction of the FERM domain with PtdInsP significantly reduces the association of SNX27 with endosomal recycling compartments (Ghai et al., 2015). Further studies revealed that an exposed β-hairpin in the PDZ domain of SNX27 in rats engages a groove in the arrestin-like structure of VPS26 (Gallon et al., 2014). This binding of SNX27 and VPS26 increases the affinity of the SNX27 PDZ domain for PDZ-binding motifs by an order of magnitude, revealing cooperation in cargo selection (Gallon et al., 2014). Indeed, cargoes such as NPxY/NxxY and a large group of G-protein coupled receptors (GPCRs) were trafficked in an SNX27-dependent manner from EEs to SNX-BAR-retromer-decorated tubules (Joubert et al., 2004; Lunn et al., 2007; Lauffer et al., 2010; Balana et al., 2011; Ghai et al., 2011; Temkin et al., 2011; Steinberg et al., 2013).

### Wiskott-Aldrich Protein and SCAR Homolog (WASH) Complex

In addition to SNX27, the mammalian retromer complex recruits another primary protein complex called WASH, which contributes to precise trafficking of cargoes out of endosomes (Gomez and Billadeau, 2009; Harbour et al., 2010; Cullen and Carlton, 2012). The WASH complex was initially identified through affinity isolation of tagged WASH1 protein. The WASH complex is comprised of five proteins: KIAA1033 (also known as strumpellin and WASH-interacting protein (SWIP)), strumpellin, FAM21, WASH1, and coiled-coil domain containing 53 (CCDC53; Figure 1; Derivery et al., 2009; Gomez and Billadeau, 2009). Each WASH complex component is unstable and degrades if the other subunits are silenced in mammalian cells (Derivery et al., 2009; Jia et al., 2010; Seaman et al., 2013). These results strongly support the hypothesis that the proteins operate as an obligate complex in which they are dependent on each other (Gomez et al., 2012). FAM21 binds retromer through its unstructured C-terminal tail, which contains numerous repeated motifs called LFa motifs, and binds to the surface of endosomes in mammalian cells (Figure 1; Harbour et al., 2012). The two most C-terminal LFa motifs, R20 and R21, bind to the VPS35/VPS29 complex with the highest affinity; endosomal localization of FAM21 is abolished when R20 and R21 are deleted in mammalian cells (Jia et al., 2012; Hao et al., 2013). In addition, VPS35-VPS29 interaction is also critical for regulation of retromer association with the WASH complex; the H675R mutation in human VPS35 blocks binding to VPS29 and prevents association with FAM21 and the WASH complex (Helfer et al., 2013). The results described above suggest that CSC recruits the WASH complex to the endosomal surface.

The WASH complex and activated Arp2/3 facilitate actin polymerization and mediate patch formation on endosomes (Derivery et al., 2009; Gomez and Billadeau, 2009). Discrete actin patches may provide a platform for specialized signaling events; rapid elongation of actin filaments can generate a localized force that may be involved in the formation and maturation of endosomal tubules through which internalized transmembrane proteins are generally thought to exit the cell (Maxfield and McGraw, 2004; Bonifacino and Rojas, 2006). Finally, Mvp1 promotes VPS1-mediated fission of retromer and coats tubules that bud from the endosome (Chi et al., 2014). Therefore, WASH plays a major role in retromer-dependent endosomal trafficking.

Recent studies have extended the CSC interactome (Steinberg et al., 2013), but very few CSC interactomes have been subjected to comprehensive study; therefore, further research is needed to thoroughly study all CSC interactomes. Some molecules reported to interact with retromer or its accessory proteins play significant roles in various neurodegenerative diseases.

## Retromer and Neurodegenerative Diseases

### Alzheimer’s Disease

AD is a major cause of dementia in the developed world. Abnormal accumulation of neurotoxic amyloid beta (Aβ) peptides produced by cleavage of amyloid precursor protein (APP) is the hallmark of AD (Small and Duff, 2008). APP can be processed through amyloidogenic and non-amyloidogenic pathways. At the PM, most APP molecules are cleaved by β-secretase, releasing soluble APP (sAPP). However some precursor molecules escape this non-amyloidogenic pathway and are internalized via the endocytic pathway, following trafficking from endosomal compartments to the TGN or endolysosomes. Within endosomal compartments and the TGN, APP is sequentially cleaved by β-secretase (BACE) and γ-secretase, resulting in formation of Aβ peptides of varying lengths, including Aβ40 and Aβ42 (Choy et al., 2012).

In 2005, reduced protein levels of VPS35 and VPS26 were found in the entorhinal cortex of brains from AD patients, leading to increased focus on the role of retromer in AD pathogenesis. In mice, VPS35 haploinsufficiency increases Aβ protein levels, whereas deficiencies in the retromer-sorting pathway increase the length of time that APP resides in the endosomes; both effects can be linked to late-onset AD. In flies, disruption of retromer function decreases secretion of Aβ40, increasing the Aβ42:Aβ40 ratio and causing Aβ to aggregate into neurotoxic oligomers and amyloid plaques, leading to hippocampal-dependent memory deficits and synaptic dysfunction (Small et al., 2005; Wen et al., 2011; Bhalla et al., 2012; Vardarajan et al., 2012; Muhammad et al., 2008; Sullivan et al., 2011). Therefore, retromer may take part in APP metabolism, possibly by influencing trafficking of APP from the endosomes to the TGN (Choy et al., 2012). Direct binding between APP and the retromer complex has not been reported (Andersen et al., 2005; Wen et al., 2011); however, several proteins that participate in APP trafficking and processing are dependent on retromer trafficking, including sortilin-related receptors SORLA and SORCS1, BACE1, and some phagocytosis receptors related to Aβ (Gustafsen et al., 2013).

BACE1 and γ-secretase contribute critically to abnormal APP metabolism. VPS35 dysfunction in the mouse hippocampus increased BACE1 activity, whereas suppression of BACE1 expression rescued VPS35 deficiency (Wen et al., 2011; Wang et al., 2012). Suppression of VPS35 or VPS26 expression in cultured cells decreased BACE1 trans-Golgi localization (Wen et al., 2011). The VPS10p domain-sorting receptor sortilin is also required for retromer-mediated retrieval of BACE1 from endosomes to the TGN (Tan and Evin, 2012). Deletion of the intracellular domain of sortilin, containing the putative retromer-binding domain, increased endosomal localization of sortilin and BACE1 (Lane et al., 2012). In addition, interfering with SNX6 expression increased BACE1-dependent secretion of sAPPand cell-associated fragment C99, as well as Aβ generation (Okada et al., 2010). With respect to γ-secretase, SNX27 physically binds to PS1, a subunit of γ-secretase, and disrupts the integrity of the complex, thus inhibiting APP proteolysis (Wang et al., 2014). These results demonstrate the crucial function of the retromer complex in suppression of AD neuropathology and inhibition of BACE1 activation and Aβ production, which are mediated by promoting BACE1 endosome-to-Golgi retrieval.

**Figure 2:**
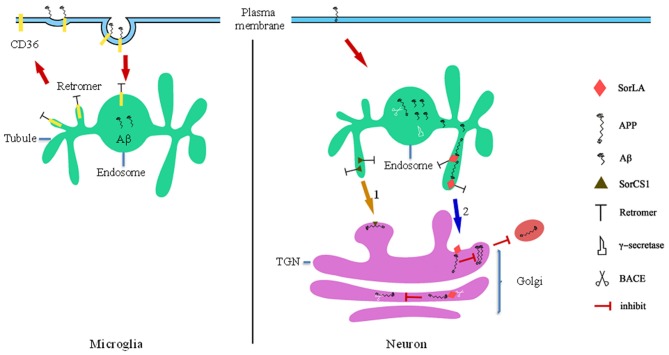
**Retromer-mediated sorting in Alzheimer’s disease (AD).** In microglia, retromer regulates the recycling pathway of phagocytic receptors to the cell surface for reuse, such as CD36 which can promote Aβ clearance. Retromer dysfunction decreases the recycle efficiency of phagocytic receptors, which leads to microglia abnormalities. BACE is response to cleave the amyloid precursor protein (APP) to Aβ (of varying lengths), and BACE is more activity in endosomes compartments because of low PH. In neuron, retromer retrievals its sorting receptor SorLA and SorLA from endosomes to TGN. (1) SorCS1 modulates retrograde TGN trafficking of APP from endosomal compartments resulting in increased APP localization to the TGN and decreased Aβ. (2) SorLA functions in slowing APP exit from the TGN into the secretory pathway whereby interacts with APP blocks the ability of APP to form homodimers, which are the preferred substrates of secretase. Secondly, SORL1 acts as a molecular link between APP and BACE and inhibits BACE interacts with APP in Golgi, which reduces cleavage of APP.

SorLA/LR11 belongs to a family of VPS10-containing receptors and is expressed in neurons. Transcription of SORL1, the gene encoding SORLA, is down-regulated in patients suffering from sporadic AD. In addition, SORLA interacts directly with APP in N2a cells (Scherzer et al., 2004; Andersen et al., 2005; Schmidt et al., 2007; Mehmedbasic et al., 2015). Ablation of sortilin expression in mice results in accumulation of Aβ in the brain, implying that low SorLA expression may be a risk factor for developing AD. SorLA slows the exit of APP from the TGN into the secretory pathway, inhibiting the formation of APP homodimers, the preferred substrates of secretase (Figure 2; Rogaeva et al., 2007; Schmidt et al., 2012). SORL1 acts as a molecular link between APP and BACE and inhibits the interaction between BACE and APP in the Golgi apparatus, reducing APP cleavage (Figure 2). However, SorLA only inhibits APP processing when located in the TGN, where its CR-cluster is essential for binding; deletion of the CR-cluster abolishes the protective effects of SorLA against APP processing (Fjorback and Andersen, 2012; Mehmedbasic et al., 2015), indicating that SorLA functions as a TGN retention factor for APP. Retromer mediates retrograde transport of SorLA through direct interaction between VPS26 and the cytoplasmic tail of SorLA; when this interaction is disrupted, SorLA can be transported to the TGN and interact with newly synthesized APP (Fjorback and Andersen, 2012). Consequently, overexpression of SorLA may reduce the abundance of Aβ and inhibit Aβ aggregation, perhaps slowing the progression of neurodegeneration associated with AD.

SorCS1 is a VPS10 domain-containing protein that shows significantly reduced expression levels in amygdala from AD brains. In cultured cells, SorCS1 interacts with APP and regulates Aβ generation (Reitz et al., 2011). In the brains of wild-type mice, SorCS1 forms a complex with APP, SorL1, and VPS35 (Lane et al., 2013). In the brains of female SorCS1-deficient mice, VPS35 abundance is decreased, while Aβ abundance is increased, providing further evidence of the role of SorCS1 in regulating Aβ generation and implicating retromer in such regulation (Lane et al., 2010). Initially, reduced SorCS1 expression facilitated APP processing by γ-secretase (Reitz et al., 2011). SorCS1 modulates retrograde TGN trafficking of APP and/or APP C-terminal fragments (CTF) from endosomal compartments (Figure 2); its cytoplasmic tail motif YAQM facilitated APP localization to the TGN and decreased Aβ production in H4 neuroglioma cells (Lane et al., 2013). Therefore, within low pH endosomal compartments, especially in LEs, APP is cleaved by BACE and γ-secretase, followed by formation of Aβ (Xu, 2009). However, sorting of SorCS1 from endosomes to the TGN is dependent on retromer (Figure 2; Hermey, 2009; Lane et al., 2013). In addition, retromer may also take part in SorCS1-dependent endosome-to-lysosome trafficking of APP/APP CTFs. The results described above demonstrate that low expression levels of SORLA and SORCS1 causes pathogenic processing of APP by decreasing the time that APP resides in the TGN; therefore, we hypothesize that increasing expression of SORLA and SORCS1 or facilitating trafficking of these proteins from endosomes to the TGN might have protective effects against AD.

Apart from impacting APP processing, recent studies found that microglia from the brains of human patients with AD exhibit reduced retromer expression and a decreased rate of Aβ phagocytosis. Phagocytosis receptors, including CD36, tripartite motif containing 2 (Trim2), and class A1 scavenger receptors (Scara1), function in the clearance of extracellular proteins, including Aβ (Prada et al., 2006; Frenkel et al., 2013; Lucin et al., 2013). During the process of phagocytosis, phagocytosis receptors interact with ligands and are taken into the cell. Subsequently, phagocytosis receptors are recycled to the PM for reuse (Freeman and Grinstein, 2014). Therefore, to some extent, the recycling efficiency of the phagocytosis receptor controls the clearance efficiency of its ligand. Decreased retromer expression impacts the recycling efficiency of CD36 and Trim2 from endosomes to the PM, inhibiting Aβ clearance (Figure 2; Lucin et al., 2013). The results described above suggest that recovering the abundance of retromer in the microglia of AD patients may enhance phagocytic efficiency and induce removal of Aβ aggregates.

Other AD risk genes, including SNX1, SNX3, and RAB7A, are involved in the cargo-selective retromer complex, demonstrating the direct link between the activity of the retromer complex and the pathogenesis of AD (Vardarajan et al., 2012).

### Parkinson’s Disease

PD is the second most common neurodegenerative disease worldwide after AD. PD is associated with two main pathological hallmarks: loss of pigmented dopaminergic (DA) neurons in the substantia nigra pars compacta and the presence of Lewy bodies composed primarily of α-synuclein (αSYN) fibrils (Spillantini et al., 1997).

Utilizing exosome sequencing, two independent research groups identified the same mutation in the VPS35 gene (P.D620N) as the first disease-causing gene for PD (Vilariño-Güell et al., 2011). In the following years, the D620N mutation in VPS35 was identified, leading researchers to focus on this mutation as the probable disease-causing pathogenic gene in autosomal dominant familial or late-onset autosomal dominant PD patients (Vilariño-Güell et al., 2011; Zimprich, 2011; Ando et al., 2012). Overexpression of VPS35 or VPS26 significantly protected VPS35^DAT-Cre^ mice from locomotor deficits, while increasing the shortened life span of *Drosophila* serving as a model of PD (Linhart et al., 2014; Miura et al., 2014; Tang et al., 2015). In addition, VPS35 protein suppressed αSYN expression in a prion-like seeding model in transgenic mice, protecting the mice from neurodegeneration (Linhart et al., 2014; Ross et al., 2015). Finally, the retromer complex can mask the toxic effects of alterations in levels of translation initiation factor expression (Ross et al., 2015). These results suggest that retromer plays a crucial role in the development of PD.

**Figure 3:**
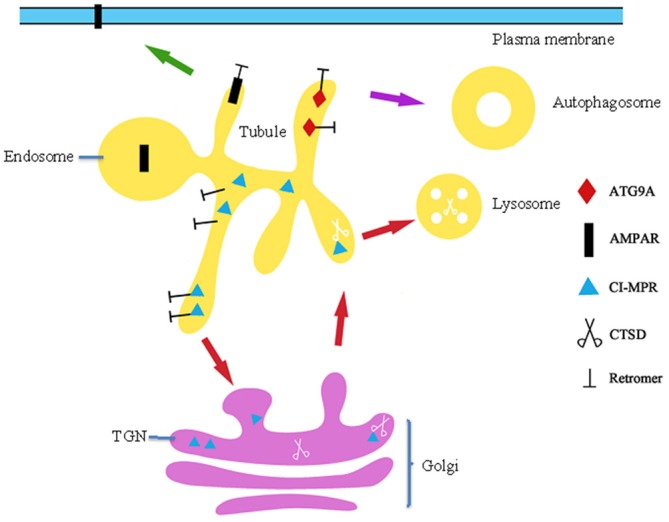
**The role of retromer in trafficking of AMPAR, ATG9A and CI-MPR.** Firstly, retromer is response for recycle of AMPA type glutamate receptor (AMPAR) to the PM that mediates vast majority of excitatory transmission with CNS and excitatory synaptic transmission. Secondly, Retromer in charge of transport autophagy-related protein ATG9A to the autophagosomes, mutation p.D620N affects ATG9A localization and trafficking results in impaired autophagosomes formation. Finally, retromer retrieves unoccupied CI-MPRs in endosomes and traffics them to the TGN, where they take part in cycles of CTSD to the lysosomes.

Two studies suggest that loss of DA neurons in patients with PD is caused by mitochondrial dysfunction. In DA neurons, VPS35 deficiency, loss, or mutation (P.D620N) increased the half-life and protein level of mitochondrial E3 ubiquitin ligase-1 (MUL1) and led to mitofusin-2 (MFN2) ubiquitination and degradation, thus leading to mitochondrial fragmentation and DA neuron loss (Tang et al., 2015). Another study verified that mitochondrial deficits in neurons were caused by P.D620N-enhanced interaction of the dynamin-like protein 1 (DLP1) complex with VPS35, resulting in abnormal transport of DLP1 to the lysosomes for degradation (Wang et al., 2016). DLP1 is as an inhibitor of mitochondrial fission events (Chan, 2006). Synaptic dysfunction may also lead to neuronal death. The p.D620N mutation inhibits recycling of AMPA-type glutamate receptors (AMPARs), which mediate the vast majority of excitatory transmission within CNS to the PM (Figure 3). Such perturbations to synaptic function likely produce chronic pathophysiological stress upon neuronal circuits that may contribute to neurodegeneration in this form, and other forms of Parkinsonisms (Munsie et al., 2015). The studies described above demonstrate that retromer defects lead to neuron death.

The pathogenic oligomers formed by various intermediate states of αSYN are suspected to be the toxic species underlying PD. FcγRIIB contributes to phagocytosis of αSYN into the cell (Wales et al., 2013; Choi et al., 2015). Aspartyl protease cathepsin D (CTSD), a lysosomal enzyme, is responsible for degradation of long-lived αSYN (Cullen et al., 2009). Under physiological conditions, cation-independent mannose-6-phosphate receptor (CI-M6PR) is mainly localized in the TGN and LEs in the perinuclear space. Newly synthesized CTSD binds CI-M6PR in the TGN and is subsequently translocated into endosomes (Figure 3). Once at the endosomes, CTSD is released from CI-M6PR and awaits the next opportunity for transport to the lysosomes, at which point it becomes active (Figure 3; Miura et al., 2014). Retromer retrieves unoccupied CI-M6PRs from endosomes and traffics them to the TGN, where they take part in further cycles of CTSD sorting (Figure 3). However, p.D620N leads to a perturbation of CI-M6PR in endosome-to-TGN transport and results in a more scattered distribution of CI-M6PR in the periphery, markedly impacting processing of CTSD into the mature 20 kDa active form and thus leading to abnormal αSYN accumulation (Follett et al., 2014; McGough et al., 2014; Zavodszky et al., 2014). Silencing of VPS35 in cultured cells also reduced the distribution of CI-MPR and impairs maturation of CTSD concomitant with striking accumulation of αSYN in the lysosomes, which may contribute to the pathogenesis of PD (Miura et al., 2014). Autophagosomes have been implicated as an intracellular site in which aggregations of αSYN are cleared. In 2012, Dengjel used quantitative proteomics to identify VPS35 as a stimulus-dependent interacting partner of autophagosomes in human breast cancer cells, while a more recent study demonstrated that VPS35 directly interacts with and retrieves Atg8 in *Magnaporthe oryzae* (Dengjel et al., 2012). These studies suggest that retromer is closely related to the formation of autophagosomes in mammalian cells. Because p.D620N poorly binds with the WASH complex and impairs recruitment of WASH to endosomes, the affinity of p.D620N/VPS29 to R20 and R21 was significantly lower than that of the wild-type VPS35/VPS29 (McGough et al., 2014). This poor affinity affects autophagy-related protein ATG9A trafficking (Figure 3), while localization to the autophagosome impairs autophagosome formation, perhaps causing accumulation of αSYN (Zavodszky et al., 2014; Munsie et al., 2015). Although VPS26A and VPS29 interact with VPS35 in the CSC in the retromer complex, only two rare nonsynonymous variants (VPS26A, p.K93E; and VPS29, p.N72H) were identified in 702 PD patients, suggesting that mutations in VPS26A and VPS29 are not a frequent cause of PD (Koschmidder et al., 2014; Shannon et al., 2014; Gustavsson et al., 2015). VPS35 interacts genetically and functionally with translation initiator EIF4G1, a PD gene and scaffold protein on which the translation initiation complex assembles (Dhungel et al., 2015; Ross et al., 2015). Expression of VPS35 or sortilin proteins can suppress toxicity associated with EIF4G1 up-regulation, masking the toxic effects of alterations in levels of translation initiation factor expression (Dhungel et al., 2015).

The studies mentioned above suggest that malfunction of VPS35 plays a primary role in the pathogenesis of PD, implying that overexpression of VPS35 may inhibit the development of PD and extend the lifespan of PD patients.

### Other Neurodegenerative Diseases

Retromer participates in the pathogenesis of neurological disorders other than AD and PD. In hereditary spastic paraplegia, three disease-causing mutations, V626F, L619F, and N471D, have been described in highly conserved sequences of strumpellin. Mutant strumpellin increases the vulnerability of corticospinal neurons, a pathological feature of hereditary spastic paraplegia (Freeman et al., 2013). In Down syndrome caused by over-expression of miR-155, a chromosome 21-encoded micro RNA negatively regulates C/EBPβ, a transcription factor that modulates SNX27 expression. Reduced SNX27 expression impacts glutamate receptor recycling to the PM in the hippocampus and produces grossly abnormal neuroanatomy, leading to synaptic dysfunction and deficits in learning and memory (Wang et al., 2013).

Endosomal transport of various pathogens, such as *Salmonella, Herpesvirus saimiri, Coxiella burnetii*, human papilloma virus, *Legionella pneumophila*, and Shiga toxins, is also mediated by the CSC (Bujny et al., 2007, 2008; Popoff et al., 2007; Kingston et al., 2011; Finsel et al., 2013; McDonough et al., 2013). Several pathogens interact directly with retromer, but the manner in which retromer interacts with many of its substrates is unknown.

### Conclusions and Future Perspectives

The trend in pathophysiological research on neurodegenerative diseases is shifting from studying disease-specific, causative, misfolded proteins towards determining the pathways and protein complexes that may contribute to the pathogenesis of such diseases.

Interestingly, most neurodegenerative diseases have a close relationship with retromer complex defects (reduced expression or mutations). Reduced retromer levels were found in several parts of the brains of patients with AD or PD, suggesting that reduced retromer abundance may have been associated with these conditions. Therefore, future studies should determine the potential of retromer as a therapeutic target for treatments intended for patients with neurological disorders.

In patients with AD, PD, and prion diseases, inhibition of microglial phagocytosis results in aggregation of Aβ, αSYN, and prion protein (PrP^Sc^; Ciesielski-Treska et al., 2004; Lucin et al., 2013; Choi et al., 2015). Several studies found that inhibition of microglial phagocytosis by defects in various receptors and proteins that participated in phagocytosis resulted in neurodegeneration and amyloidosis in mouse models of AD (Lu and Lemke, 2001; Wyss-Coray et al., 2002; Kaifu et al., 2003; Heneka et al., 2013). In contrast, microglial activation reduced Aβ pathology in mouse models of AD (Heneka et al., 2013). Retromer is critically involved in recycling phagocytosis receptors, including CD36, Trem2, and CED-1, to the PM for reuse; such recycling is vital for the clearance of misfolded proteins and cellular debris (Chen et al., 2010; Lucin et al., 2013; Abduljaleel et al., 2014). Increased abundance of retromer facilitated phagocytosis and reduced Aβ plaque formation in the brains of APP transgenic mice by increasing the efficiency of phagocytosis receptor recycling (Lucin et al., 2013). These studies highlight the importance of retromer-mediated phagocytosis in brain homeostasis and suggest that retromer may represent a therapeutic target for the treatment of neurological diseases.

Retromer may also be a therapeutic target for treatments intended to relieve neuronal damage associated with neurodegenerative diseases. VPS35-deficiency leads to neuron loss in Tg2576 mice and DA neuron loss in young adult mice (Wen et al., 2011). However, deficient mitochondrial dynamics associated with neurodegenerative disorders is a factor in neuron loss (Tang et al., 2015). VPS35 plays a role in the formation of mitochondria-derived vesicles, suggesting that mitochondria may be a potential target site for VPS35 function. Expression of wild-type WVPS35 in VPS35-deficient cells restored mitochondrial morphology and function in DA neurons, as well as mitochondrial fission dynamics in fibroblasts (Tang et al., 2015; Wang et al., 2016). VPS35 suppressed MUL1-mediated MFN2 degradation and promoted mitochondrial fusion (Züchner et al., 2004; Tang et al., 2015). However, MFN2-loss is implicated in the pathogenesis of Charcot-Marie-Tooth disease type 2A (Züchner et al., 2004). Altered mitochondrial morphology and dynamics were detected in VPS35-deficient DA neurons, as well as VPS35-depleted neuroblastoma and fibroblast cell lines (Tang et al., 2015; Wang et al., 2016). These results suggest that VPS35 may be a general regulator of mitochondrial fusion and fission dynamics *in vitro*, indicating that it could be a therapeutic target for treatments intended to prevent neuron loss in patients with neurodegenerative diseases.

In addition to the diseases mentioned above, retromer also plays a role in Creutzfeldt-Jakob disease (CJD), diabetes, and optic nerve injury (Kipkorir et al., 2014; Liu et al., 2014; Morabito et al., 2014). Future studies should determine why substantially decreased retromer expression was found in several parts of the brains of patients with the diseases mentioned above, but not in the whole brain. In addition, future studies should attempt to determine why retromer dysfunction occurs in specific neuronal populations. Comprehensive clinical studies are required to determine the potential of retromer as a therapeutic target.

## Author Contributions

CL wrote the manuscript. SZAS reviewed and edited the manuscript. LY and DZ provided the idea and gave useful advise during all major steps of writing the manuscript. All authors have read and approved the final manuscript.

## Conflict of Interest Statement

The authors declare that the research was conducted in the absence of any commercial or financial relationships that could be construed as a potential conflict of interest.
